# Association of Serum Immunoglobulins Levels With Specific Disease Phenotypes of Crohn's Disease: A Multicenter Analysis in China

**DOI:** 10.3389/fmed.2021.621337

**Published:** 2021-04-28

**Authors:** Dong Juan Song, Jun Shen, Min Hu Chen, Zhan Ju Liu, Qian Cao, Pin Jin Hu, Xiang Gao, Jia Ming Qian, Kai Chun Wu, Li Jie Lai, Zhi Hua Ran

**Affiliations:** ^1^Division of Gastroenterology and Hepatology, Key Laboratory of Gastroenterology and Hepatology, Ministry of Health, Inflammatory Bowel Disease Research Center, Renji Hospital, School of Medicine, Shanghai Jiao Tong University, Shanghai Institute of Digestive Disease, Shanghai, China; ^2^Department of Gastroenterology, The First Affiliated Hospital of Sun Yat-sen University, Guangzhou, China; ^3^Department of Gastroenterology, The Shanghai Tenth People's Hospital of Tongji University, Shanghai, China; ^4^Department of Gastroenterology, Sir Run Run Shaw Hospital, College of Medicine, Zhejiang University, Hangzhou, China; ^5^Department of Gastroenterology, The Sixth Affiliated Hospital of Sun Yat-sen University, Guangzhou, China; ^6^Department of Gastroenterology, Peking Union Medical College Hospital, Peking Union Medical College, Chinese Academy of Medical Sciences, Beijing, China; ^7^Department of Gastroenterology, Xijing Hospital, Air Force Military Medical University, Xi'an, China

**Keywords:** Crohn's disease, disease phenotypes, disease activity, serum immunoglobulins, cross-sectional study

## Abstract

**Background and Aim:** Serum immunoglobulins were reported to be associated with clinical characteristics of inflammatory bowel disease. However, whether a difference exists in the serum immunoglobulins levels in patients with Crohn's disease (CD) with different disease location and behavior phenotypes remains unclear. Therefore, this study aimed to explore the associations of serum immunoglobulins levels with specific CD phenotypes.

**Methods:** Patients with CD having recorded serum immunoglobulins levels were recruited through multicenter collaborative efforts. The associations between serum immunoglobulins levels and distinct phenotypes of CD were evaluated using multiple logistic regression models.

**Results:** A total of 608 patients with CD were included in the study. Elevated (above the upper limit of normal) serum immunoglobulin G (IgG), IgA, IgM, and IgG4 were identified in 24.5, 17.4, 2.1, and 8.2% of patients, respectively. Elevated serum IgG4 levels negatively correlated with complicated disease behavior [odds ratio (OR) 0.49, 95% confidence interval (CI) 0.26–0.92]. Elevated serum IgG was linked to isolated ileal disease with an OR of 0.37 (95% CI 0.23–0.61). The ORs of isolated ileal disease progressively reduced across increasing quartiles of IgG (*P* for trend < 0.001). The adjusted ORs of isolated ileal disease for increasing quartiles of IgM were 1.82 (1.07–3.1), 1.92 (1.14–3.24), 1.17 (0.69–1.98), and 1 (*P* for trend = 0.008). Besides, serum IgA and IgG levels significantly correlated with several disease activity indices.

**Conclusions:** These results suggested that certain serum immunoglobulins were associated with specific disease phenotypes of CD. Further investigations to account for the associations are warranted.

## Introduction

Crohn's disease (CD), one subtype of inflammatory bowel disease (IBD), is a chronic, relapsing-remitting inflammatory disorder involving the gastrointestinal tract. The increased prevalence of IBD with time in developing nations has been confirmed (1, 2). The innate and adaptive immune systems are critical to the development of IBD, and the latter is the more proximate driver of tissue damage in patients with IBD (3). The adaptive immune system is composed of B cells, T cells, and regulatory T/B cells. The function of B cells in the etiology of IBD has received increasing attention in recent years (4, 5). B cells are transformed into plasma cells, which synthesize and release immunoglobulin G (IgG), IgA, IgM, IgD, and IgE. The isotypes and subclasses of immunoglobulins have distinct effector functions and represent particular immunologic processes (6). The change in the serum levels of immunoglobulin isotypes has been confirmed and the distribution is unique in different autoimmune diseases, thus confirming the role of certain isotypes of immunoglobulin in disease development (7–9). Serum IgG has been found to provide risk prediction in patients with immunoglobulin A nephropathy, autoimmune hepatitis accompanied by systemic lupus erythematosus (SLE-AIH), and hepatitis B virus-related acute-on-chronic liver failure (HBV-ACLF) (10–12).

Serum immunoglobulins levels have been reported to be associated with distinct clinical characteristics of IBD. Previous studies showed that the levels of serum IgG, IgG1, and IgG4 were significantly increased in ulcerative colitis (UC) compared with CD. Conversely, the serum IgG2 levels were significantly decreased in UC compared with CD (13, 14). In addition, a possible relationship between low serum IgG or IgG1 levels and the need for small bowel resection in patients with IBD has been reported (15). Furthermore, hypergammaglobulinemia defined as elevated IgG levels contributes to distinguishing arthritis from arthralgia in pediatric IBD (16). Patients with IBD having high levels of mucosal and serum IgG4 tend to have severe and extensive lesions (17).

Patients with CD can be further classified according to the Montreal classification, including age of onset, disease behavior, and disease location (18). Complicated disease (stricturing or penetrating behavior) and ileal disease location at diagnosis conferred an increased risk of intestinal resection in CD (19, 20). Thus, patients with CD with specific disease phenotypes may benefit from early intervention. Nevertheless, studies focusing on the associations of serum immunoglobulins levels with different disease location and behavior phenotypes of CD are limited. A clear understanding of the immune mechanisms involved in CD contributes to risk stratification and personalized prevention.

It was hypothesized that the expression of serum immunoglobulins is different in patients with CD with different disease location and behavior phenotypes. Hence, a cross-sectional study was performed to explore the possible associations between serum immunoglobulins levels and phenotypic features of CD.

## Methods

### Patient Population

Patients with CD with recorded serum immunoglobulins levels between 2016 and 2018 were recruited from seven tertiary hospitals in China. The diagnosis of CD was performed according to the European Crohn's and Colitis Organization (ECCO) consensus (21). Meanwhile, patients with CD coexisting with other autoimmune diseases were excluded. Finally, the present study comprised 608 individuals who received serum IgG, IgA, IgM, and IgG4 testing.

The study was approved by the Medical Ethics Committee of Renji Hospital, School of Medicine, Shanghai Jiao Tong University.

### Clinical Characteristics of CD Patients

The demographic data, Montreal classification of CD, surgical history, history of appendectomy and perianal operation, disease activity, and other laboratory tests such as C-reactive protein (CRP), erythrocyte sedimentation rate (ESR), hemoglobin, platelet (PLT) count, albumin, and prealbumin were collected from each patient. CD phenotypes were evaluated based on the Montreal classification. Complicated disease was defined as B2, B3, and B2B3 (22). Perianal disease consisted of perianal abscess and perianal fistula. Surgical history was bowel resection associated with CD. The disease activity was determined based on the Harvey-Bradshaw index (23).

### Analysis of the Levels of Serum IgG, IgA, IgM, and IgG4

As a retrospective study using electronic medical records, the serum immunoglobulins assays used were not controlled. The serum immunoglobulins levels were considered as elevated when they were higher than the upper limit of normal in each hospital. The serum immunoglobulins levels from different centers were normalized based on their distributions for quantitative and correlation analyses (24–26).

### Statistical Analysis

All statistical analyses were carried out using SPSS software (version 19). Categorical variables were presented as numbers (percentages) and continuous variables as medians [interquartile range (IQR)] unless specifically annotated. The normal distribution of continuous data was determined using the Kolmogorov-Smirnov test. Comparisons of percentages between groups were performed using the chi-square or Fisher's exact test. Comparisons between groups for ordered variables were performed using the rank-sum test. Statistical differences for normally distributed data between two groups were analyzed using the Student *t-*test. Non-normally distributed continuous variables were analyzed using non-parametric tests. Spearman's correlation test was applied for data with non-normal distribution. Laboratory data from different hospitals were normalized according to their distribution as mentioned earlier. Principal component analysis (PCA) was performed with the R Statistic program version 4.0.2.

The multivariate logistic regression was used to assess the independent associations between serum immunoglobulins levels and the presence of complicated disease and isolated ileal disease. The serum immunoglobulins levels were also categorized into quartiles to determine the shapes of relationship. These confounders in the models were selected according to their relationship with dependent variables or a change in the effect estimate of more than 10%. Tests for linear trend were conducted by entering the median values of each category as a continuous variable. *P-*values of <0.05 indicated a statistically significant difference.

## Results

### Patient Characteristics

The baseline characteristics of participants are shown in Table 1. Among the total of 608 patients, 68.4% were male and the median age of the included patients was 30.5 years (IQR 25–39 years). Isolated ileal disease was observed in 42.3% of participants. Complicated disease was observed in 60.4% of participants. The PCA analysis for data collected from different centers revealed there was no obvious separation from our centers to other centers (Supplementary Figure 1).

**Table 1:** Baseline characteristics of the study population.

	**All patients (*n* = 608)**
Age, y, median (IQR)	30.5 (25–39)
Age at diagnosis, y, median (IQR)	29 (23–37)
Male, *n* (%)	416 (68.4%)
**Disease duration (y)**, ***n*** **(%)**	
≤1	179 (29.4%)
1–5	228 (37.5%)
5–10	138 (22.7%)
>10	63 (10.4%)
**Disease location**, ***n*** **(%)**	
L1 ± L4	257 (42.3%)
L2 ± L4	50 (8.2%)
L3 ± L4	301 (49.5%)
**Disease behavior**, ***n*** **(%)**	
B1: Inflammatory	241 (39.64%)
B2: Stricturing	265 (43.58%)
B3: Penetrating	47 (7.73%)
B2B3: Stricturing + Penetrating	55 (9.05%)
Perianal disease, *n* (%)	328 (53.9%)
Complicated disease, *n* (%)	367 (60.4%)
Appendectomy, *n* (%)	51 (8.4%)
Perianal operation, *n* (%)	178 (29.3%)
Surgical history, *n* (%)	100 (16.4%)
Treatment naïve patients	192 (31.6%)
Active/remission	537/71

*y, years; IQR, interquartile range*.

### Demographic and Clinical Data of Patients in Accordance With the Serum Immunoglobulins Status

Elevated serum IgG, IgA, IgM, and IgG4 were found in 24.5, 17.4, 2.1, and 8.2% of participants, respectively. The univariate analysis was not performed for IgM because only 13 of 608 patients had elevated serum IgM levels. The univariate analysis of demographic and phenotypic characteristics associated with elevated serum IgG, IgA, and IgG4 are listed in Table 2. Elevated serum IgG was related to gender (*P* = 0.015), age (*P* = 0.011), disease duration (*P* = 0.033), history of appendectomy (*P* = 0.011), surgical history (*P* = 0.001), disease location (*P* < 0.001), and disease activity (*P* = 0.004). Elevated serum IgA was significantly related to age (*P* = 0.009), age at diagnosis (*P* = 0.026), disease location (*P* = 0.003), disease behavior (*P* = 0.007), and disease activity (*P* < 0.001). Elevated serum IgG4 was related to age (*P* < 0.001), age at diagnosis (*P* < 0.001), disease duration (*P* < 0.001), and complicated disease (*P* = 0.006).

**Table 2:** Demographic and phenotypic characteristics of cases according to serum total IgG, IgA, and IgG4 status.

	**Elevated IgG**	**Normal IgG**	**P**	**Elevated IgA**	**Normal IgA**	**P**	**Elevated IgG4**	**Normal IgG4**	***P***
	**(*n* = 149)**	**(*n* = 459)**		**(*n* = 106)**	**(*n* = 502)**		**(*n* = 50)**	**(*n* = 558)**	
Male	90 (60.4%)	326 (71%)	0.015	66 (62.3%)	350 (69.7%)	0.13	36 (72%)	380 (68.1%)	0.57
Age, Median (IQR)	29 (22–38)	31 (25–39)	0.011	28 (24–34.25)	31 (25–40)	0.009	24 (19–32.25)	31 (25–39)	<0.001
Age at diagnosis, Median (IQR)	27 (21–36)	29 (23–37)	0.067	27 (22–32)	29 (23–38)	0.026	23 (19–31.25)	29 (23–37)	<0.001
Disease duration, Median (IQR)	2 (0.75–6)	3.3 (1–7)	0.033	3 (0.96–7)	3 (1–7)	0.24	1 (0.5–3)	3.5 (1–7)	<0.001
Appendectomy	5 (3.4%)	46 (10%)	0.011	12 (11.3%)	39 (7.8%)	0.23	1 (2%)	50 (9%)	0.15
Perianal operation	42 (28.2%)	136 (29.6%)	0.74	26 (24.5%)	152 (30.3%)	0.24	16 (32%)	162 (29%)	0.66
Surgical history	12 (8.1%)	88 (19.2%)	0.001	15 (14.2%)	85 (16.9%)	0.48	4 (8%)	96 (17.2%)	0.09
Disease Location			<0.001			0.003			0.54
L1 ± L4	31 (20.8%)	226 (49.24%)		30 (28.3%)	227 (45.2%)		24 (48%)	233 (41.76%)	
L2 ± L4	20 (13.4%)	30 (6.53%)		14 (13.2%)	36 (7.2%)		5 (10%)	45 (8.06%)	
L3 ± L4	98 (65.8%)	203 (44.23%)		62 (58.5%)	239 (47.6%)		21 (42%)	280 (50.18%)	
Isolated ileal disease	31 (20.8%)	226 (49.2%)	<0.001	30 (28.3%)	227 (45.2%)	0.001	24 (48%)	233 (41.8%)	0.39
Disease behavior			0.068			0.007			0.049
B1: Inflammatory	69 (46.3%)	172 (37.5%)		39 (36.8%)	202 (40.2%)		29 (58%)	212 (38%)	
B2: Stricturing	51 (34.2%)	214 (46.6%)		38 (35.8%)	227 (45.2%)		14 (28%)	251 (45%)	
B3: Penetrating	14 (9.4%)	33 (7.2%)		11 (10.4%)	36 (7.2%)		3 (6%)	44 (7.9%)	
B2 B3: Stricturing	15 (10.1%)	40 (8.7%)		18 (17%)	37 (7.4%)		4 (8%)	51 (9.1%)	
and penetrating									
Complicated disease	80 (53.7%)	287 (62.5%)	0.055	67 (63.2%)	300 (59.8%)	0.51	21 (42%)	346 (62%)	0.006
Perianal disease	89 (59.7%)	239 (52.1%)	0.103	64 (60.4%)	264 (52.6%)	0.14	29 (58%)	299 (53.6%)	0.55
Disease activity^a^			0.004			<0.001			0.99

a *Comparison of disease activity was performed using Rank-sum test*.

### Independent Association of Serum IgG4 Levels With Complicated Disease

**Figure 1:**
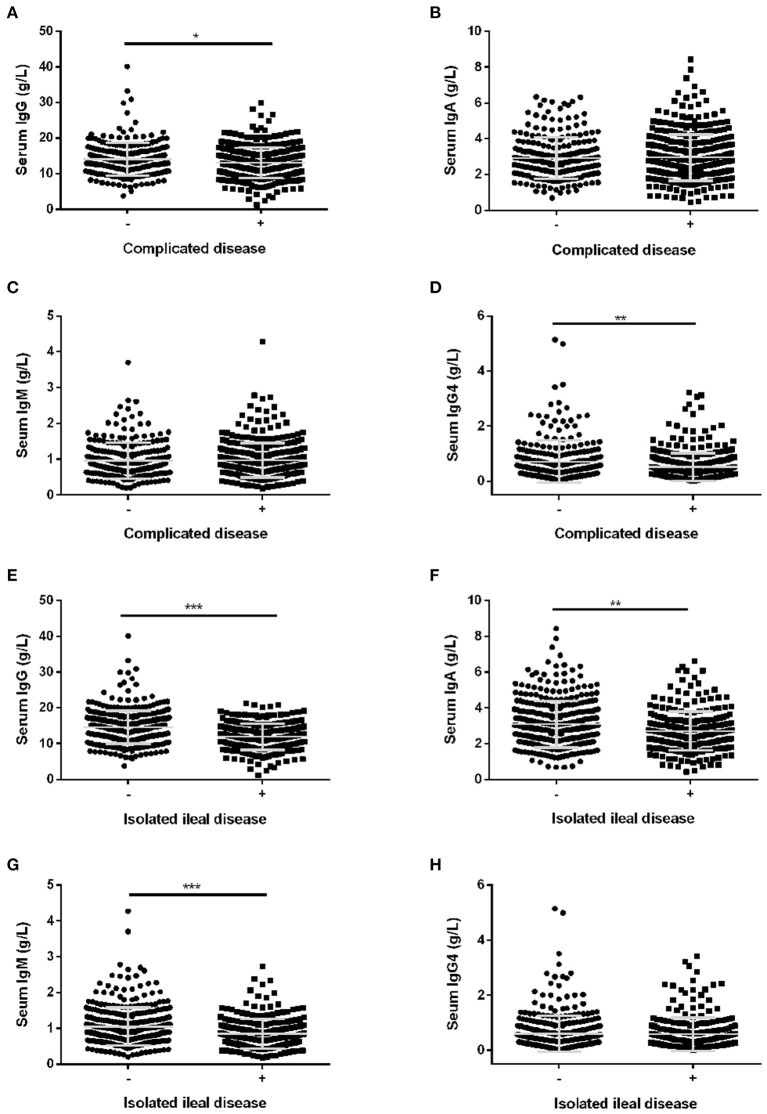
The levels of serum immunoglobulins in CD patients with different disease phenotypes. The levels of serum IgG **(A)**, serum IgA **(B)**, serum IgM **(C)**, and serum IgG4 **(D)** in CD patients with complicated disease. The levels of serum IgG **(E)**, IgA **(F)**, IgM **(G)** and IgG4 **(H)** in CD patients with isolated ileal disease. Bar graphs are presented as mean ± SD. *P < 0.05, **P < 0.01, ***P < 0.001.

The levels of serum IgG and IgG4 were significantly reduced in CD patients with complicated disease compared with CD patients with inflammatory phenotype (Figures 1A,D). However, there was no difference in serum IgA and IgM levels in CD patients with and without complicated disease (Figures 1B,C). Univariate analysis revealed that elevated serum IgG4 was inversely related to complicated disease (OR: 0.44, 95% CI: 0.25–0.8) (Table 3). Multiple logistic regression was employed to further evaluate the association of serum IgG4 with complicated disease. When serum IgG4 levels were assessed as quartiles, a significantly increased probability of complicated disease was found in patients in quartile 3 (OR: 1.82, 95% CI: 1.11–2.98) and quartiles 1–2 (OR: 1.86, 95% CI: 1.2–2.88) compared with those in quartile 4. For a per-standard deviation (SD) increase in serum IgG4 levels, the OR for complicated disease was 0.74 (0.62–0.89) in the final multivariable model (Table 3).

**Table 3:** Association of serum IgG4 levels with complicated disease.

	**Crude**	**Model 1^a^**	**Model 2^b^**
Elevated serum IgG4	0.44 (0.25–0.8)	0.49 (0.27–0.89)	0.49 (0.26–0.92)
Quartile 1 and quartile 2 of serum IgG4	1.92 (1.29–2.85)	1.82 (1.21–2.74)	1.86 (1.2–2.88)
Quartile 3 of serum IgG4	1.66 (1.05–2.63)	1.59 (1.002–2.52)	1.82 (1.11–2.98)
Quartile 4 of serum IgG4	Reference	Reference	Reference
P for trend	0.001	0.004	0.005
Per-SD increase	0.72 (0.61–0.86)	0.74 (0.62–0.88)	0.74 (0.62–0.89)

*Data are odds ratio (95% confidence interval)*.

a *Adjusted for gender and age*.

b *Adjusted for gender, age, disease duration, history of appendectomy and intestinal surgery, disease location, and disease activity*.

### Independent Association of Serum IgG and IgM Levels With Isolated Ileal Disease

In accordance with the results of univariate analysis in Table 2, the levels of serum IgG were significantly reduced in CD patients with isolated ileal disease (Figure 1E). Similar tendencies were also observed for IgA and IgM (Figures 1F,G). However, there was no difference in serum IgG4 levels in CD patients with and without isolated ileal disease (Figure 1H). Furthermore, the crude and adjusted associations of serum IgG and IgM with isolated ileal disease were analyzed by multivariate logistic regression and are presented in Tables 4, 5, respectively. Elevated serum IgG was negatively related to isolated ileal disease (OR: 0.37, 95% CI: 0.23–0.61). The OR of isolated ileal disease was 1 (95% CI: 0.61–1.63) for quartile 2, 0.55 (95% CI: 0.33–0.92) for quartile 3 and 0.3 (0.165–0.53) for quartile 4 compared with quartile 1 in the final multivariate model (*P* for trend < 0.001). An SD increase in serum IgG levels was associated with a 45% reduction in the adjusted probability of isolated ileal disease. Besides, the adjusted ORs between decreasing quartiles of serum IgM levels and the presence of isolated ileal disease were as follows: 1, 1.17 (0.69–1.98), 1.92 (1.14–3.24), and 1.82 (1.07–3.1) (*P* for trend = 0.008). An SD increase in serum IgM levels was related to isolated ileal disease with an adjusted OR of 0.74 (95% CI: 0.6–0.91). In all, serum IgG and IgM levels had a negative correlation with isolated ileal disease.

**Table 4:** Association of serum IgG levels with isolated ileal disease.

**Serum IgG**	**Crude**	**Model 1^a^**	**Model 2^b^**
Elevated serum IgG	0.27 (0.175–0.42)	0.29 (0.185–0.45)	0.37 (0.23–0.61)
Quartile 1 of serum IgG	Reference	Reference	Reference
Quartile 2 of serum IgG	0.83 (0.53–1.31)	0.835 (0.53–1.32)	1 (0.61–1.63)
Quartile 3 of serum IgG	0.44 (0.28–0.7)	0.46 (0.29–0.74)	0.55 (0.33–0.92)
Quartile 4 of serum IgG	0.2 (0.12–0.33)	0.21 (0.13–0.355)	0.3 (0.165–0.53)
*P* for trend	<0.001	<0.001	<0.001
Per-SD increase	0.485 (0.4–0.59)	0.5 (0.41–0.61)	0.55 (0.43–0.7)

*Data are odds ratio (95% confidence interval)*.

a *Adjusted for gender, age at diagnosis*.

b *Adjusted for gender, age at diagnosis, history of appendectomy and intestinal surgery, serum IgA, and IgM, perianal disease, complicated disease, and disease activity*.

**Table 5:** Association of serum IgM levels with isolated ileal disease.

**Serum IgM**	**Crude**	**Model 1^a^**	**Model 2^b^**
Quartile 1 of serum IgM	2.97 (1.845–4.78)	2.5 (1.525–4.1)	1.82 (1.07–3.1)
Quartile 2 of serum IgM	2.47 (1.535–3.97)	2.145 (1.32–3.49)	1.92 (1.14–3.24)
Quartile 3 of serum IgM	1.52 (0.94–2.46)	1.4 (0.85–2.28)	1.17 (0.69–1.98)
Quartile 4 of serum IgM	Reference	Reference	Reference
*P* for trend	<0.001	<0.001	0.008
Per-SD increase	0.62 (0.51–0.75)	0.67 (0.55–0.815)	0.74 (0.6–0.91)

*Data are odds ratio (95% confidence interval)*.

a *Adjusted for gender, age at diagnosis*.

b *Adjusted for gender, age at diagnosis, history of appendectomy and intestinal surgery, serum IgA and IgG, perianal disease, complicated disease, and disease activity*.

### Correlation of Serum IgA and IgG Levels With Disease Activity

**Figure 2:**
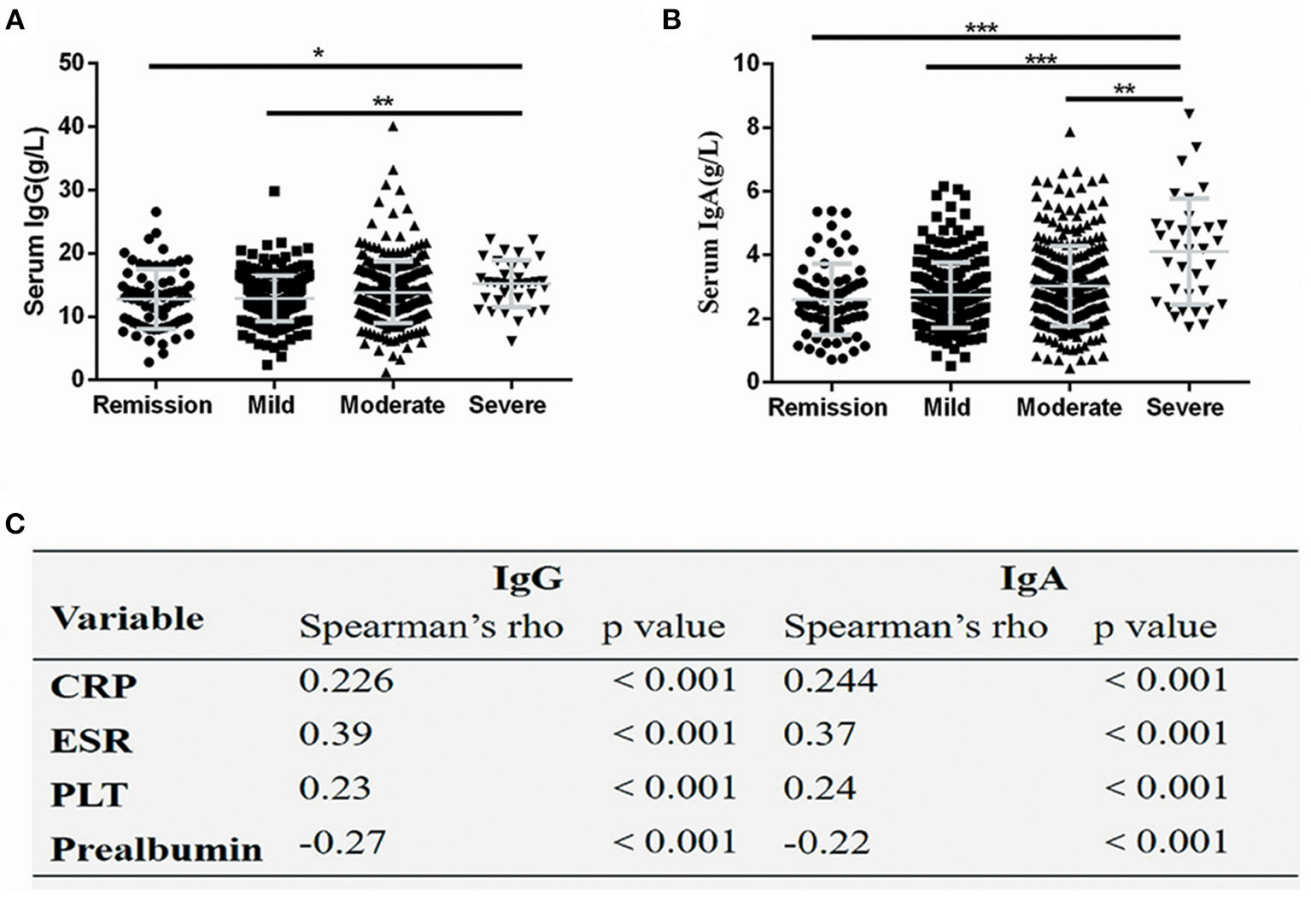
Serum IgG and IgA levels correlated with disease activity. Serum IgG **(A)** and IgA **(B)** values were significantly increased in severely active Crohn's disease, The spearman coefficient of correlation between serum IgG and IgA and several markers of disease activity **(C)**. The symbols represent values in individual patients. Values are shown as mean ± SD. *P < 0.05, **P < 0.01, ***P < 0.001. CRP, C-reactive protein; ESR, erythrocyte sedimentation rate; PLT, platelet count.

Further, the serum immunoglobulins levels in CD patients with different disease activities were compared. The serum IgG levels were significantly lower in CD patients in remission and patients with mild disease compared with patients with severe disease (*P* < 0.05) (Figure 2A). The serum IgA levels were significantly decreased in CD patients in remission and patients with mild-to-moderate disease compared with patients with severe disease (*P* < 0.05) (Figure 2B). The correlation analysis revealed that serum IgG and IgA levels positively correlated with CRP and ESR levels, and PLT count, but negatively correlated with prealbumin levels (*P* < 0.001) (Figure 2C).

## Discussion

This study evaluated the associations between serum immunoglobulins levels and complicated disease phenotype, isolated ileal disease, and disease activity of CD. The results indicated that serum IgG4 levels inversely correlated with complicated disease behavior. Serum IgG and IgM levels were found to be negatively associated with isolated ileal disease. In addition, serum IgA and IgG levels correlated with the disease activity of patients with CD.

The clinical implications of abnormal serum IgG4 levels in IBD patients has been explored with inconsistent results. A recent study conducted in United States has revealed that IBD patients with low serum IgG4 levels were more likely to experience complicated disease progress such as increased rates of CD-related surgeries and IBD-related hospitalizations as well as increased requirement for biologics, systemic steroids, and antibiotics (27). However, another study conducted in China revealed that elevated serum or mucosal IgG4 levels were associated with more extensive disease in patients with IBD (17). In our study, a negative association between elevated serum IgG4 levels and the presence of complicated disease was observed. Reduced interleukin (IL)-10 production in complicated disease may be responsible for low serum IgG4 levels in complicated disease. The IL-10 production of whole blood cell cultures was significantly reduced in CD patients with stricturing and penetrating phenotype compared with CD patients with inflammatory phenotype (28). Furthermore, IL-10 has been found to enhance IgG4 production by IL-4-stimulated peripheral blood mononuclear cells and was suggested to potentiate both IgG4 switching and growth of IgG4-secreting B cells (29, 30). However, further mechanistic studies are needed to account for this phenomenon.

The present study also found that serum IgG levels negatively correlated with isolated ileal involvement. Signatures of gut microbiota in CD patients with ileal disease were distinct from those in CD patients with colonic and ileocolonic disease. CD patients with isolated ileal disease had stronger microbial dysbiosis (31), which may be an important mechanism to induce the reduced serum IgG levels. Substantial studies have revealed that microbiota and microbial products indeed regulate serum IgG levels, although the precise mechanism is unclear yet. Serum IgG levels were significantly reduced in germ-free mice compared with conventional mice (32). Under homeostatic conditions, a selective subset of gram-negative symbiotic bacteria possesses the ability to disseminate systemically to induce systemic IgG responses, which is dependent on T cells and Toll-like receptor 4 on B cells. And one possible mechanism is that symbiotic bacteria possess additional virulent factors that help them to enter the bloodstream and thereby activate systemic immune response (33). In addition, probiotics supplementation including *Bifidobacterium bifidum, Lactobacillus frumenti*, and *Bacillus subtilis* can increase serum IgG levels in hosts such as infants, piglets and rabbits, respectively (34–36). Furthermore, short-chain fatty acids, products of the fermentation of dietary fibers by intestinal microbiota, can augment systemic IgG responses through regulating gene expression for plasma B cell differentiation and increasing cellular metabolism as well as boosting glycolytic activity in B cells (37). However, further investigations are required to explain the exact mechanism underlying the association between serum IgG levels and isolated ileal involvement.

The clinical implications of abnormal serum IgG in different clinical settings are inconsistent. Low serum IgG has been reported to be associated with poor outcomes in patients with sepsis and septic shock (38) as well as patients with immunoglobulin A nephropathy (10). However, elevated serum IgG may predict poor prognosis in patients with SLE-AIH (11) and in patients with HBV-ACLF (12). Our cross-sectional study suggested CD patients with isolated ileal disease had lower serum IgG levels. Whether there is a potential link to the study conducted in United States that showing IBD patients with low serum IgG/G1 levels required more small bowel resections (15) remains to be further explored. In addition, more longitudinal studies exploring the clinical significance of abnormal serum IgG levels in patients with IBD are warranted.

IgM has been found to be an anti-inflammatory factor and elevated IgM levels indicated a better immune status (39). It has been reported that reduced plasma IgM levels at the onset of sepsis predicted decreased sepsis survival (40). In our cohort, CD patients with isolated ileal involvement were more likely to have low serum IgM levels. Similar to serum IgG, increasing evidence suggests that serum IgM levels can also be influenced by microbiota. Lysozyme supplementation could alter the composition of gut microbiota and increase serum IgM levels in sows, and correlation coefficients revealed that *Ruminiclostridium 9* significantly positively correlated with serum IgM levels (39). Additionally, a positive relationship between serum IgM levels and *Bacteroidetes/Firmicutes* ratio has been reported in healthy middle-aged people (41). Besides, a positive correlation was also found between total IgM level and the abundance of *Synergistetes* in patients with SLE (42). Furthermore, reduced serum IgM level correlated with the HLA-DRB1^*^03 allelic variant among patients with SLE and controls (43). These findings suggested that IgM levels might be partially influenced by gut microbiota and genetic factors. As to IBD, HLA^*^DRB1^*^04 and DQB1^*^04 were linked to only small intestine involvement in patients with CD (44). HLA DRB1^*^0103 was suggested to be associated with colonic disease in IBD (45). Thus, in our study, we speculate that the variation in serum IgM levels in different location phenotypes might be partially due to the alteration of gut microbiota or heredity.

The correlation analysis in our study revealed that serum IgA and IgG levels significantly correlated with CRP, ESR, PLT count and prealbumin level, consistent with a study reporting that serum IgA levels had a positive correlation with several markers of disease activity in CD patients (13). However, another study reported that serum IgG and IgA levels were not associated with disease activity of CD and UC, but serum IgG and IgA levels were significantly increased in patients with pouchitis and cuffitis (46). Previous studies have demonstrated that IgA and IgG coating could identify pathogenic bacteria in IBD (47, 48). Furthermore, the levels of fecal soluble IgA and IgG were positively associated with disease activity in patients with IBD, and the correlation between soluble IgA or IgG in feces and CRP or ESR was stronger than our results (49), probably due to the difference in the type of sample analyzed. In clinical practice, IgG and IgA levels in feces are probably superior to those in serum for identifying the degree of disease activity in CD patients due to its non-invasive nature and stronger correlation.

This study has several limitations. It was cross-sectional in design. Repeated measurements and longitudinal observation of serum immunoglobulins are required to evaluate alteration of serum immunoglobulins levels along with change in disease phenotypes of CD. On the other hand, despite adjustments for some potential confounding factors, the possibility that other factors not included in the study might influence the results could not be ruled out. Smoking, alcohol consumption, and common metabolic abnormalities have been found to be related to serum levels of immunoglobulins in an adult population (50). However, these factors were not included in the present study. In addition, healthy controls were not recruited in this study. Although serum immunoglobulins levels in the background population could not be provided, the results of this study were compared with those of other studies and few differences were observed. Elevated serum IgG was identified in 24.5% of participants in the present cohort, which was comparable with a study that found 23% of pediatric CD patients had elevated serum IgG levels (16). Furthermore, 8.2% of patients in the present study had increased serum IgG4 levels, which was higher than the 6.34% reported in four kinds of autoimmune diseases, including primary Sjogren syndrome, systemic sclerosis, SLE, and primary biliary cirrhosis (9) and lower than the 9.9% reported in IBD patients in Sichuan, China (17). Finally, the normal reference ranges of serum immunoglobulins, as well as other laboratory tests such as CRP, ESR, PLT, and prealbumin from different centers were not consistent. Although these data were normalized before quantitative and correlation analyses, the application of a central laboratory was more perfect.

More prospective longitudinal studies are required to reveal whether the abnormal serum immunoglobulins participate in the disease progress of different phenotypes of CD or just are the consequence of disease development. It has been reported that CD patients with different location phenotypes have different natural history and should be regarded as separate groups. It would be worth exploring whether CD patients with abnormal serum immunoglobulins levels showing unique clinical trajectory. Furthermore, the studies involving the clinical implications of the abnormal serum IgG4 levels in patients with IBD from different races showed different results (17, 27). Thus, whether the results from a region can be transferred to another region is worthy of future research. Collectively, identifying the causality between serum immunoglobulins levels and specific disease phenotypes of CD and the underlying mechanisms may contribute to risk stratification and personalized prevention for patients with CD.

## Conclusion

In summary, the present study provided a novel insight into the associations of serum immunoglobulins with different phenotypes of CD. Our findings suggested that CD patients with complicated disease phenotype are more likely to have reduced serum IgG4 levels and CD patients with isolated ileal disease are more likely to have low serum IgG and IgM levels. In addition, serum IgA and IgG levels positively correlated with disease activity in patients with CD. Further studies are required to elucidate the pathogenic mechanism linking serum immunoglobulins levels with different phenotypes of CD.

## Data Availability Statement

The raw data supporting the conclusions of this article will be made available by the authors, without undue reservation.

## Ethics Statement

The studies involving human participants were reviewed and approved by Medical Ethics Committee of Renji Hospital, School of Medicine, Shanghai Jiao Tong University. Written informed consent for participation was not required for this study in accordance with the national legislation and the institutional requirements.

## Author Contributions

DS: data collection, statistical analysis, and manuscript drafting. JS: study design and critical revision of the manuscript. MC, ZL, QC, PH, XG, JQ, and KW: data collection and review of the article. LL: data collection and statistical analysis. ZR: study design, data interpretation and critical revision of the manuscript. All authors contributed to the article and approved the submitted version.

## Conflict of Interest

The authors declare that the research was conducted in the absence of any commercial or financial relationships that could be construed as a potential conflict of interest.

## Supplementary Material

Supplementary Figure 1 Principal components analysis (PCA) plot of clinical data based on centers.
