# Supplementary figures and images for: Association of Serum Immunoglobulins Levels With Specific Disease Phenotypes of Crohn's Disease: A Multicenter Analysis in China
Source: Front Med (Lausanne). 2021 Apr 28;8:621337. doi: 10.3389/fmed.2021.621337 (PMC8115723; doi:10.3389/fmed.2021.621337)

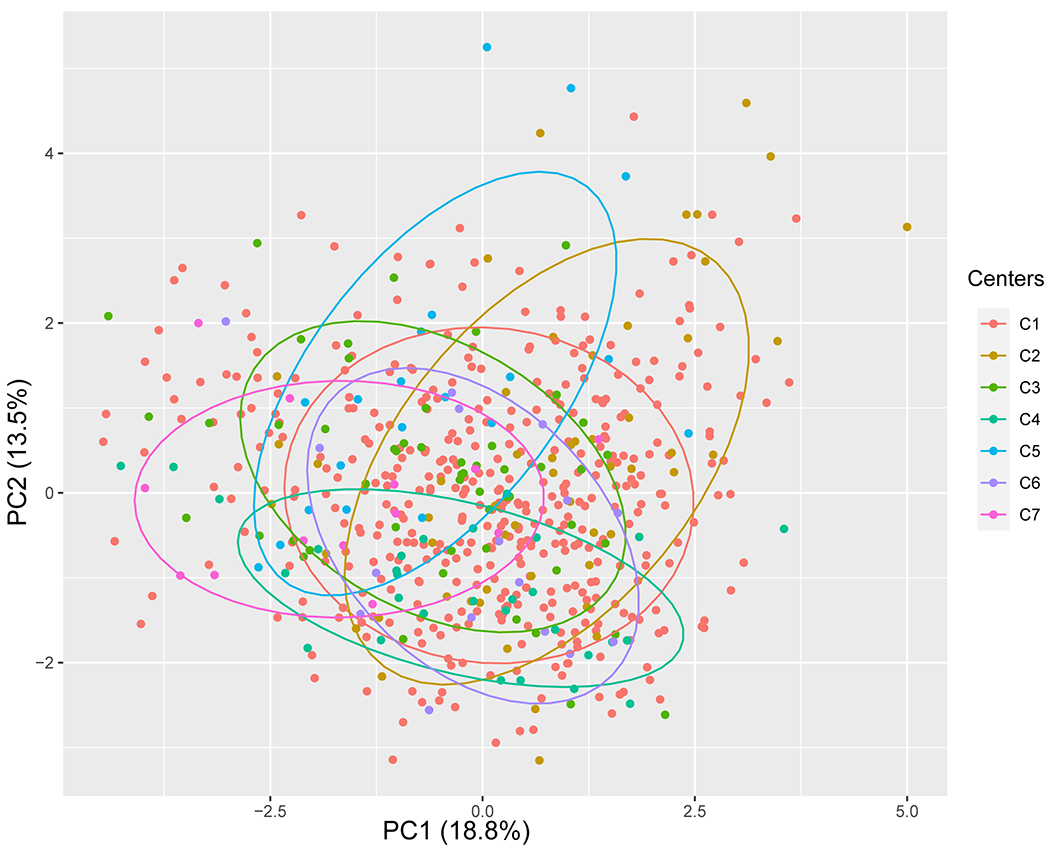

Supplement: Supplementary Figure 1 — Principal components analysis (PCA) plot of clinical data based on centers. [file Image_1.TIF]
